# Functional Distinctions of Endometrial Cancer-Associated Mutations in the Fibroblast Growth Factor Receptor 2 Gene

**DOI:** 10.3390/cells12182227

**Published:** 2023-09-07

**Authors:** Garima Dixit, Benjamin A. Pappas, Gourav Bhardwaj, Willow Schanz, Thorsten Maretzky

**Affiliations:** 1 Inflammation Program and Division of Infectious Diseases, Department of Internal Medicine, Roy J. and Lucille A. Carver College of Medicine, University of Iowa, Iowa City, IA 52242, USA; garima-dixit@uiowa.edu (G.D.); benjamin-pappas@uiowa.edu (B.A.P.); schanzwillow@gmail.com (W.S.); 2 Fraternal Order of Eagles Diabetes Research Center and Division of Endocrinology and Metabolism, Department of Internal Medicine, Roy J. and Lucille A. Carver College of Medicine, University of Iowa, Iowa City, IA 52242, USA; gourav-bhardwaj@uiowa.edu; 3 Immunology Graduate Program, Roy J. and Lucille A. Carver College of Medicine, University of Iowa, Iowa City, IA 52242, USA; 4 Holden Comprehensive Cancer Center, Roy J. and Lucille A. Carver College of Medicine, University of Iowa, Iowa City, IA 52242, USA

**Keywords:** a disintegrin and metalloprotease 17 (ADAM17), fibroblast growth factor receptor 2 (FGFR2), epidermal growth factor receptor (EGFR), heparin-binding EGF-like growth factor (HB-EGF), endometrial cancer (EC)

## Abstract

Functional analysis of somatic mutations in tumorigenesis facilitates the development and optimization of personalized therapy for cancer patients. The *fibroblast growth factor receptor 2* (*FGFR2*) gene is frequently mutated in endometrial cancer (EC), but the functional implications of *FGFR2* mutations in cancer development remain largely unexplored. In this study, we introduced a reliable and readily deployable screening method to investigate the effects of *FGFR2* mutations. We demonstrated that distinct mutations in *FGFR2* can lead to differential downstream consequences, specifically affecting a disintegrin- and metalloprotease 17 (ADAM17)-dependent shedding of the epidermal growth factor receptor (EGFR) ligand heparin-binding EGF-like growth factor (HB-EGF) and phosphorylation of mitogen-activated protein kinases (MAPKs). Furthermore, we showed that the distribution of mutations within the *FGFR2* gene can influence their oncogenic effects. Together, these findings provide important insights into the complex nature of *FGFR2* mutations and their potential implications for EC. By unraveling the distinct effects of different mutations, our study contributes to the identification of personalized treatment strategies for patients with *FGFR2*-mutated cancers. This knowledge has the potential to guide the development of targeted therapies that specifically address the underlying molecular alterations associated with *FGFR2* mutations, ultimately improving patient outcomes in EC and potentially other cancer types characterized by *FGFR2* mutations.

## 1. Introduction

The advent of high-throughput genome sequencing has provided unprecedented opportunities to interrogate the mutational landscape of cancers. This effort has resulted in significant insights into the genetic underpinning and evolution of human cancer [1,2,3]. Deeper exploration of these cancer-associated mutations has identified a subset of driver mutations critical for tumorigenesis, which are distinct from passenger mutations that do not affect the fitness of cancer cells [1,2,3,4]. Endometrial cancer (EC) exhibits genetic heterogeneity, with mutations in multiple signaling pathway genes, including the FGFR2 signaling pathway, occurring in over 10% of patients [5]. Mutations in the *FGFR2* gene can impact different regions of the FGFR2 protein [6], including the extracellular domain, transmembrane domain, and intracellular domain, making FGFR2 a promising target for therapeutic interventions [7,8,9,10,11]. However, despite recognizing the importance of *FGFR2* mutations in cancers like EC [12,13], the clinical responses to FGFR inhibitors have demonstrated significant variability [14], highlighting the need for a better understanding of which FGFR2 alterations are oncogenic and can be effectively targeted therapeutically.

The FGFR2 signaling pathway has attracted our attention for several reasons. First, activated FGFR2 phosphorylates specific tyrosine residues that mediate interaction with cytosolic adaptor proteins and several downstream signaling proteins [15,16,17]. Among these, the pathway leading to activation of mitogen-activated protein kinase (MAPK) signaling has been studied most extensively [18,19,20]. Second, FGFR2-mediated phosphorylation of MAPKs is known to stimulate and to regulate the proliferation and migration of epithelial cells in the skin and endometrium [21,22,23,24,25], all of which depends on epidermal growth factor receptor (EGFR)-mediated MAPK signaling and a disintegrin and metalloprotease (ADAM) 17-dependent proteolytic release (shedding) of the EGFR ligand heparin-binding EGF-like growth factor (HB-EGF) [24,25]. Third, while many of the FGFRs can be activated by several fibroblast growth factors (FGFs), the FGFR2 is specifically activated by FGF7, making it an ideal system to study the crosstalk between FGFR2 and the EGFR signaling. Lastly, we have previously shown that the EC-linked S252W and N550K mutations in the *FGFR2* gene enhance the sensitivity to FGF7-induced activation of ADAM17 and subsequent transactivation of EGFR. Importantly, we demonstrated that the blockade of ADAM17 activity effectively inhibits FGF7-mediated oncogenic growth induced by FGFR2 in vitro and suppresses the growth of FGFR2-mutant EC in vivo [25]. These findings highlight the crucial role of ADAM17 in driving the oncogenic effects of *FGFR2* mutations in EC. In addition to S252W and N550K mutations [12], additional mutations (D101Y, K310R, C383R, I548V, and K660E) are also reported in independent EC cohorts [6,7,9]. Together, these mutations account for around 90% of all *FGFR2* mutations in EC [6,9]. Although the oncogenic nature of the S252W and N550K mutations in the *FGFR2* gene has been previously demonstrated [9], there is limited functional knowledge regarding other *FGFR2* mutations, and their oncogenic activity remains unexplored [10,26]. Additional investigations are required to determine the clinical significance of these mutations and their effects on protein function, such as activation and response to drugs. Understanding the functional characteristics of *FGFR2* mutations holds great significance in identifying potential therapeutic targets and guiding personalized treatment strategies for patients with *FGFR2* mutations in various diseases, including cancer. While targeting FGFR2 signaling shows promise as a therapeutic approach in EC, specifically in patients with FGFR2 alterations, it is crucial to acknowledge that not all EC patients have benefited from this strategy. Therefore, precise patient selection based on specific FGFR2 alterations is essential to identify individuals who are more likely to respond favorably to FGFR inhibitors.

In this study, we uncover distinct functional consequences associated with different mutations in the *FGFR2* gene. Our findings highlight the existence of two classes of *FGFR2* mutations in EC, characterized by either gain- or loss-of-function ability to activate ADAM17-mediated proteolytic ectodomain shedding and its downstream pathways. Furthermore, we underscore the utility of a cell-based screening method capable of determining the activation status of FGFR2, thus offering a valuable tool for personalized treatment decisions. The insights gained from our research shed light on the downstream mechanisms influenced by *FGFR2* mutations and carry implications for tailoring treatment options to patients with tumors harboring *FGFR2* mutations.

## 2. Materials and Methods

### 2.1. Cell Lines and Culture 

COS7 and NIH 3T3 cells (American Type Culture Collection, Manassas, VA, USA) were cultured in high-glucose Dulbecco’s modified Eagle’s medium (DMEM) supplemented with 10% heat-inactivated calf serum (FCS) and 1% penicillin/streptomycin in a humidified 5% CO_2_ incubator at 37 °C. Opti-MEM medium was used for starving the cells prior to inhibition or stimulation experiments (Thermo Fisher Scientific, Waltham, MA, USA). 

### 2.2. Growth Factors and Inhibitors

Recombinant FGF7 was obtained from R&D Systems, Minneapolis, MN, USA. The metalloprotease inhibitor DPC333 was a gift from Dr. Carl P. Blobel (Weill Cornell Medicine, Graduate School of Medical Sciences, NY, USA) and diluted in DMSO to the indicated concentrations.

### 2.3. Expression Vectors

The expression vector for the alkaline phosphatase (AP)-tagged HB-EGF has been described previously [27]. FGFR2-mutant constructs were generated by using full-length FGFR2 cDNA as a template [28]. The Quik Change site-directed mutagenesis kit (Stratagene, CA, USA) was used to generate the point mutations of FGFR2 (D101Y, S252W, K310R, C383R, I548V, N550K, and K660E).

### 2.4. Generation of Stable Clones of COS7 Cells Expressing WT and Mutant FGFR2

COS7 cells were transfected with FGFR2-WT or FGFR2-mutant plasmids at a concentration of 1.5 μg using Lipofectamine 2000 (Thermo Fisher Scientific, Waltham, MA, USA) as described [29]. Clones were isolated following zeocin (25 to 100 μg/mL) selection (Invitrogen, Carlsbad, CA, USA). Shedding results for each FGFR2 construct were confirmed in three independent clones.

### 2.5. Ectodomain Shedding Assays

Ectodomain shedding assays were performed as described earlier [25,27,30,31]. Briefly, stable clones of COS7 cells expressing WT and mutant FGFR2 growing in a 12-well plate at 65–70% confluency were transfected with Lipofectamine 2000 per the manufacturer’s protocol using AP-HB-EGF plasmids at a concentration of 1 μg per well as described [32]. For FGF7 stimulation experiments, cells were starved in Opti-MEM medium (Thermo Fisher Scientific, Waltham, MA, USA) for 1 h followed by incubations with the indicated stimulus for 45 min. Supernatants were collected and cells lysed with lysis buffer (pH 9.5) containing Tris base (100 mM), NaCl (100 mM), MgCl_2_ (20 mM), 1–10 phenanthroline (0.5 M), and EDTA (0.5 M) for 30 min at 4 °C. Constitutive shedding was measured after 2 h of incubation. Supernatants and cell lysates were loaded in triplicates on a 96-well plate and AP activity was measured by colorimetry at 405 nm. The ratio of AP activity in the supernatant to total AP activity in the cell lysate reflects the activity of ADAM17 toward AP-tagged HB-EGF. Conditioned media of non-transfected cells display no AP activity.

### 2.6. Immunoblot Analysis

For immunoblotting analysis, protein extraction from cultured COS7 cells was conducted according to previously described methods [24]. Whole-cell lysates were separated using SDS/10% polyacrylamide gels and electrophoretically transferred to a polyvinylidene fluoride membrane (Bio Trace NT Nitrocellulose Transfer Membrane, Pall Corporation, New York, NY, USA) using standard procedures. Membranes were blocked with 3% weight/volume skim milk in TBS and probed with primary antibodies against FGFR2 (1:2000 dilution, Cell Signaling Technologies, Danvers, MA, USA), phospho and total 44/42 MAPK (1:1000 dilution; Cell Signaling Technologies, Danvers, MA, USA), pFRS2 (1:500 dilution, Cell Signaling Technologies, Danvers, USA), and FRS2 (1:2000 dilution, Proteintech, Rosemont, IL, USA) followed by incubation at 4 °C for 12 h. After three washes in 0.1% Tween-TBS, the membranes were incubated with secondary antibody at 37 °C for 1 h. Peroxidase-conjugated goat anti-mouse or goat anti-rabbit antibodies (Promega, Madison, WI, USA) were used as the secondary antibodies. Immunoreactive protein bands were visualized using ECL chemifluorescent reagent (GE Healthcare, Chicago, CA, USA) and imaged with Image Studio v4.0 (LI-COR, Lincoln, USA) in the LI-COR Odyssey FC infrared imager.

### 2.7. Anchorage-Independent Growth Assays

The anchorage-independent growth assays with NIH 3T3 cells were performed as described previously [33,34]. For anchorage-independent growth assays, cells (500 in each well of a 24-well plate) were embedded in 0.3% soft agar in the presence of 5% FCS. Activators and/or inhibitors were added as indicated in figure legends. Fresh treatment solution was added at least twice a week. After 14–21 days of growth, the cell colonies were enumerated from 10 fields photographed under a bright-field microscope (EVOS XL core, Invitrogen, Carlsbad, CA, USA).

### 2.8. Statistical Analysis

All data were expressed as mean ± standard error of mean (SEM) of at least three independent experiments unless otherwise indicated and analyzed by one-way analysis of variance (ANOVA) with a Tukey test to determine group differences. Difference between two groups was investigated by an unpaired Student’s *t*-test. The analyses were performed using Prism 9.5.1 software (GraphPad Software, San Diego, CA, USA).

## 3. Results

### 3.1. Development of a Cell-Based Reporter Assay to Measure FGFR2 Activation In Vitro

**Figure 1:**
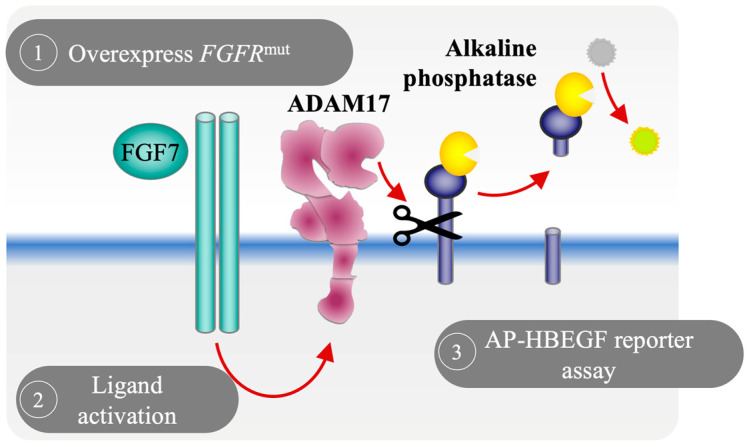
Schematic of the alkaline phosphatase assay procedure to monitor the shedding of the EGFR ligand HB-EGF in cell-based assay. Upon stimulation of a ligand, an activated FGFR2 induces ADAM17-dependent ectodomain shedding and release of membrane-bound alkaline phosphatase (AP)-HB-EGF. Released AP-HB-EGF can be quantified by measuring AP activity in the conditioned supernatants based on the production of yellow-colored *p*-NP from *p*-NPP.

To investigate whether mutations in different coding regions of the *FGFR2* gene differ in downstream consequences, we utilized a cell-based alkaline phosphatase (AP) reporter assay as a screening approach to evaluate the effects of somatic *FGFR2* mutations found in patients with EC by measuring changes in the FGFR2-activated and ADAM17-mediated release of HB-EGF [24,25]. Measuring the shedding of soluble HB-EGF poses a significant challenge due to the limited availability of antibodies for its detection. Furthermore, the released soluble HB-EGF may bind to the EGFR, thereby reducing its concentration in the cellular supernatant. These factors together make the detection of EGFR ligand shedding, such as of HB-EGF, inherently difficult. To overcome this issue, the cell-based AP reporter assay offers a reliable and consistent method to evaluate the levels of shed HB-EGF by utilizing spectrophotometric detection of AP activity in the culture supernatant (Figure 1).

**Figure 2:**
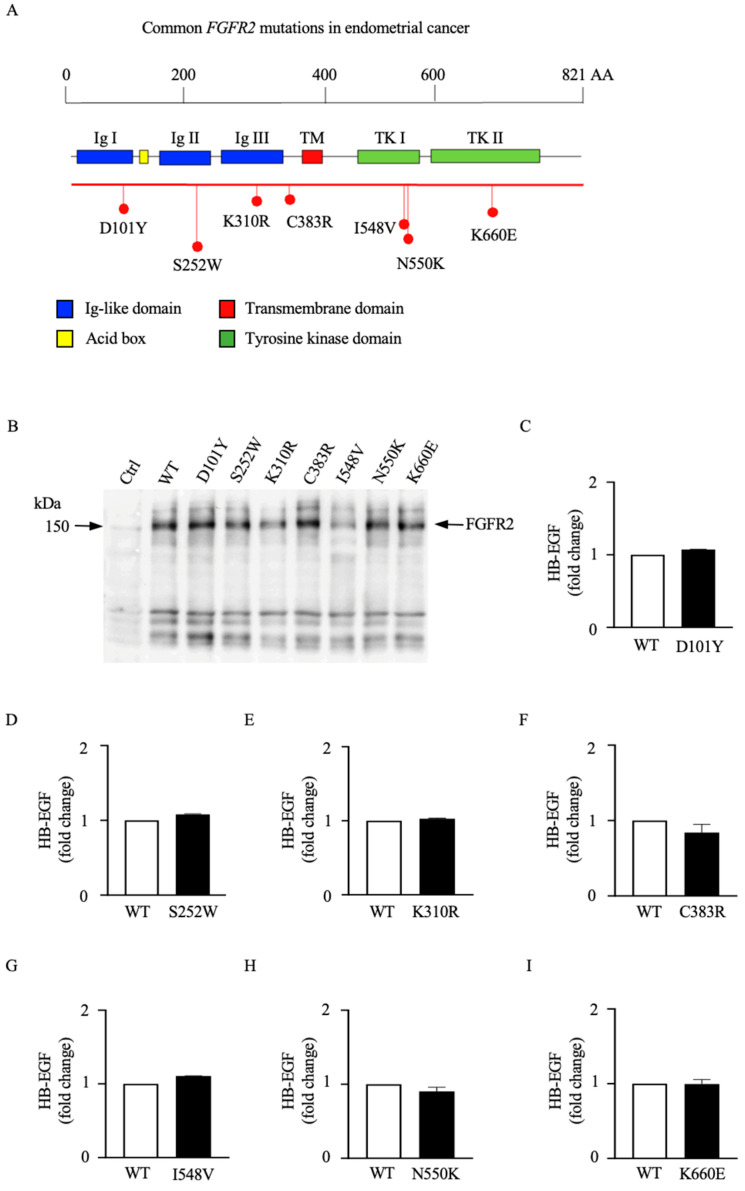
Stable overexpression of endometrial cancer mutant forms of FGFR2 does not alter the constitutive shedding activity of ADAM17 in COS7 cells. (**A**) Schematic diagram of FGFR2 protein and the point mutations introduced into *FGFR2* gene. The extracellular domain of the FGFR2 contains 3 immunoglobulin (Ig)-like domains. The intracellular sequences include a split tyrosine kinase enzyme domain (TK I and TK II). Mutations identified in [6,7] and generated for this project are mapped to functional domains. (**B**) Representative immunoblot analysis of FGFR2 expression in lysates of COS7 cells stably transfected with the generated wild type (WT) or endometrial cancer (EC) mutants of FGFR2 expression constructs. (**C**–**I**) Shedding of HB-EGF into the supernatant was analyzed by colorimetric assay for AP activity. Results are from three independent clones expressing each receptor. Data are expressed as mean ± SEM; n = 3; Welch’s *t*-test.

To evaluate the role of EC-related mutations in the *FGFR2* gene in regulating the catalytic activity of ADAM17, we introduced seven of the most commonly found point mutations in EC into full-length FGFR2 cDNA carrying expression plasmids (Figure 2A) and generated stably expressing wild type (WT) and mutant FGFR2 COS7 cell lines, which provide an isogenic background to investigate the functional impact of these mutations. Immunoblot analysis showed similar levels of full-length WT and mutant FGFR2-overexpressing COS7 cells (Figure 2B). ADAM17-mediated shedding of surface membrane proteins can occur constitutively in unstimulated cells as well as in a regulated (stimulated) fashion [35]. To examine whether stably overexpressed forms of mutant FGFR2 could affect the constitutive activity of ADAM17 in COS7 cells, we monitored the constitutive release of the EGFR ligand and ADAM17 substrate HB-EGF into the supernatant of COS7 cells stably overexpressing mutant forms of FGFR2, as previous studies utilizing *ADAM17*-deficient cells and siRNA experiments have shown that shedding of HB-EGF in endometrial cells, including EC, depends on ADAM17 activity [25]. We found that none of the tested mutations in the *FGFR2* gene affected the constitutive release of HB-EGF (Figure 2C–I). These results provide evidence that these mutations do not affect the constitutive activity of ADM17 under the conditions tested here, suggesting that responsiveness to agonist stimulation, in addition to its constitutive activity, might be required for its oncogenic activity at least in the context of ADAM17 activation.

### 3.2. Different Mutations in the FGFR2 Gene Are Functionally Distinct in Their Abilities to Activate ADAM17

**Figure 3:**
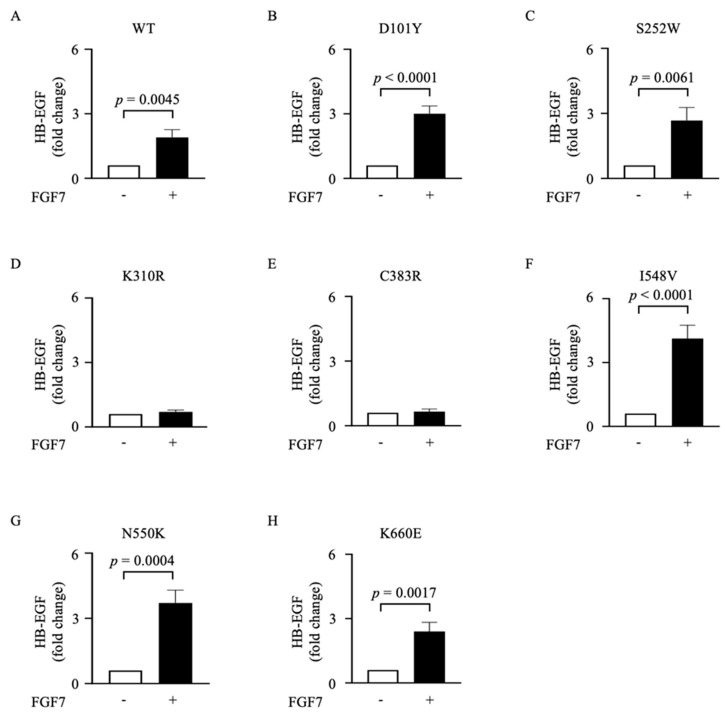
Analysis of FGF7/FGFR2-induced ADAM17-mediated release of HB-EGF in COS7 cells stably overexpressing mutant forms of FGFR2. (**A**–**H**) The ADAM17 substrate HB-EGF was transfected into COS7 cells stably overexpressing either wild type (WT) FGFR2 or mutant forms of FGFR2 carrying the specific point mutations shown in Figure 2A. Constitutive and FGF7-stimulated (50 ng/mL) shedding of the AP-tagged HB-EGF into the supernatant was measured after 45 min. Results are from three independent clones expressing each receptor, mean ± SEM; n = 3; Welch’s *t*-test. *p* values indicate significantly increased shedding in FGF7-treated (+) cells compared with vehicle-treated (-) controls.

We have previously shown that ADAM17-mediated shedding can be activated by various physiological stimuli, including thrombin, lysophosphatidic acid, and tumor necrosis factor [31]. Furthermore, previous studies have shown that growth factors, such as FGF7, stimulate the release of the EGFR ligand HB-EGF from keratinocytes and endometrial cells in a dose-dependent manner, further corroborating the critical role of ADAM17 activation in the FGF7/FGFR2 signaling axis [24,25]. When we stimulated FGFR2 mutant-expressing COS7 cells with 50 ng/mL FGF7, we observed a significant increase in shedding in the cells expressing FGFR2 (WT) as well as FGFR2 (D101Y), FGFR2 (S252W), FGFR2 (I548V), FGFR2 (N550K), and FGFR2 (K660E) but found that the FGF7-induced release of HB-EGF was abolished in mutant cell lines expressing FGFR2 (K310R) and FGFR2 (C383R) (Figure 3A–H). These findings suggest that K310R and C383R *FGFR2* mutations interfere with the ability of FGF7 to induce ADAM17-mediated HB-EGF shedding, suggesting a disruption in the signaling pathway associated with these mutations.

### 3.3. FGFR2 (S252W) Increases the Sensitivity of FGF7 Ligand-Induced ADAM17 Activity

**Figure 4:**
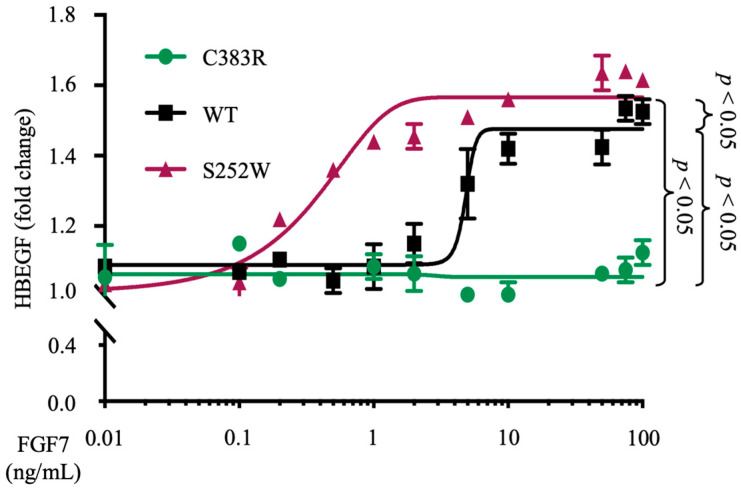
Overexpression of the somatic mutant form of FGFR2 (S252W) increases the sensitivity of FGF7/FGFR2-induced ADAM17-mediated shedding of the EGFR ligand HB-EGF. Activation of ADAM17-mediated shedding of the ADAM17 substrate and EGFR ligand HB-EGF by FGF7 was determined in COS7 cells overexpressing the S252W mutant compared with COS7 cells overexpressing wild type (WT) FGFR2 as well as with FGFR2 (C383R) as negative control. The shedding of HB-EGF in FGFR2 (S252W)-overexpressing COS7 cells was about 15 times more sensitive to FGF7 stimulation compared with COS7 cells overexpressing FGFR2 (WT). Three independent experiments were performed in triplicate. Data are expressed as mean ± SEM; two-way ANOVA showed a significant main effect of *FGFR2* gene status (*p* < 0.05).

We have previously demonstrated that S252W *FGFR2* mutant-expressing EC cell lines exhibit a reduced activation threshold in response to ADAM17 stimulation, relative to *FGFR2*-WT-expressing EC cells [25]. To determine whether this phenotype could be exclusively attributed to the mutation in *FGFR2* or if other genetic abnormalities in these cells might have played a role, we decided to investigate the most prevalent mutation of the *FGFR2* gene in EC, the S252W mutant. This particular mutation has been reported to account for more than 40% of all *FGFR2* mutations reported in EC [12]. To explore the potential of this mutation to enhance ADAM17-mediated shedding of HB-EGF through its higher affinity for the ligand FGF7, we employed an isogenic cellular system. When COS7 cells stably expressing FGFR2 (S252W), FGFR2 (C383R), or WT FGFR2 were transfected with HB-EGF and stimulated with different concentrations of the FGFR2 ligand FGF7, we observed that the shedding of HB-EGF in FGFR2 (S252W)-expressing COS7 cells (EC_50_ = 0.41 ng/mL) was about 15 times more sensitive to FGF7 stimulation compared with COS7 cells stably overexpressing WT FGFR2 (EC_50_ = 6.1 ng/mL). However, even at the highest concentration, no activation of ADAM17-mediated shedding of HB-EGF was observed in the FGFR2 (C383R)-expressing cell line (Figure 4). These findings indicate that the increased sensitivity of S252W *FGFR2* mutant-expressing EC cell lines to FGF7 stimulation, as previously observed in relation to *FGFR2* mutations, can be attributed to the specific mutation in the *FGFR2* gene present in these cells.

### 3.4. ADAM17-Dependent MAPK Phosphorylation Is Impaired in Loss-of-Function FGFR2 Mutants

**Figure 5:**
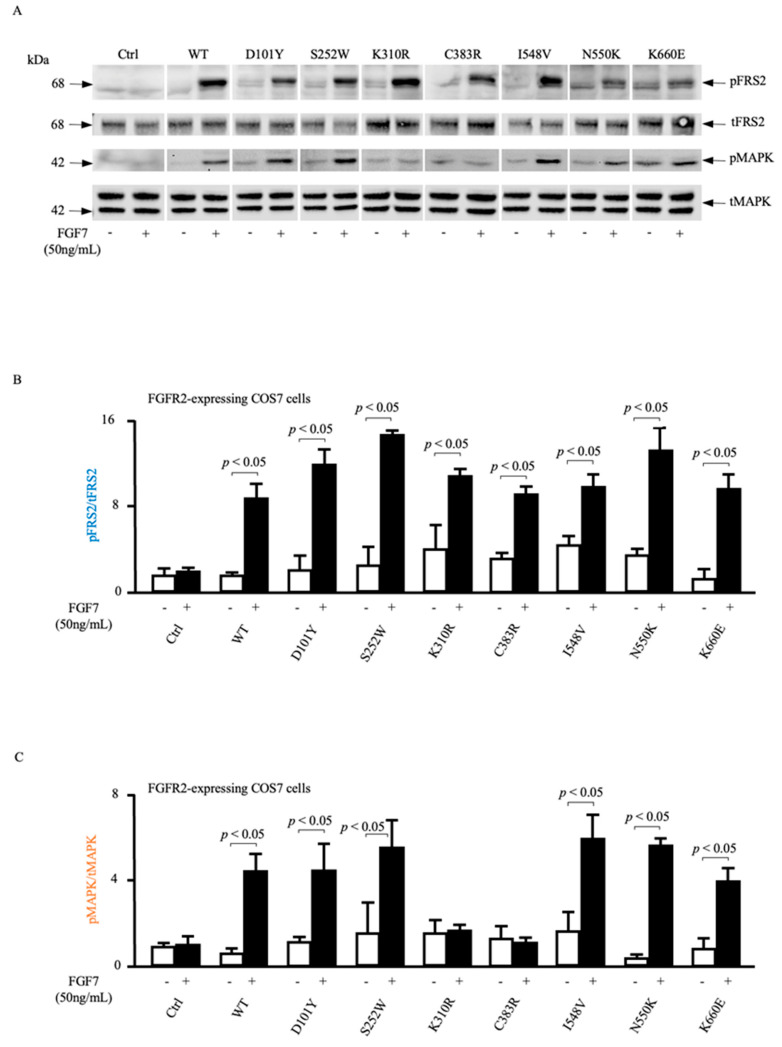
Analysis of FGF7/FGFR2-induced ADAM17-mediated phosphorylation of MAPK in COS7 cells stably overexpressing mutant forms of FGFR2. (**A**) Representative immunoblot analysis of COS7 cell lysates stably overexpressing mutant forms of FGFR2, treated with 50 ng/mL FGF7 or vehicle and blotted for phosphorylated (p) and total (t) fibroblast growth factor receptor substrate (FRS2) or pMitogen-activated protein kinase (pMAPK) and tMAPK. (**B**,**C**) Densitometric quantification of immunoblots from 3 separate experiments including those presented in A. The ratios of pFRS2 to tFRS2 (**B**) and pMAPK to tMAPK (**C**) are shown, mean ± SEM. ImageJ software was used to quantify the bands obtained via immunoblot analysis. The area under the curve (AUC) for the specific signal was corrected for the total loading control AUC. n = 3 for densitometric quantification of FRS or MAPK phosphorylation. Data are expressed as mean ± SEM; Welch’s *t*-test. *p* < 0.05 indicates significant differences in FGF7-stimulated (+) FRS2 (**B**) and MAPK (**C**) phosphorylation compared to unstimulated vehicle-treated (−) cells.

We have previously shown that FGF7-mediated stimulation of FGFR2 activates the cell surface metalloprotease ADAM17 in a Src-, PI3-kinase-, and p38 MAPK-dependent manner, which in turn leads to the ADAM17-mediated shedding of the EGFR ligand HB-EGF. This ADAM17-dependent processing of HB-EGF liberates this soluble growth factor from its membrane tether, allowing it to activate the EGFR as well as MAPK signaling and stimulate cell migration. To provide additional insights into the underlying mechanisms of mutations in the *FGFR2* gene on ADAM17-dependent activation of EGFR-mediated phosphorylation of MAPK, we evaluated the effect of FGF7 stimulation on FGFR2 activation and MAPK phosphorylation (Figure 5).

When we serum-starved COS7 cells stably overexpressing mutant forms of FGFR2 or COS7 cells expressing WT FGFR2 in response to FGF7, FGF7-dependent phosphorylation of MAPK was abolished in cells expressing FGFR2 (K310R) or FGFR2 (C383R) but not altered by WT FGFR2, FGFR2 (D101Y), FGFR2 (S252W), FGFR2 (I548V), FGFR2 (N550K), or FGFR2 (K660E). As a readout for FGFR2 activation, we assessed the phosphorylation status of fibroblast growth factor receptor substrate 2 (FRS2) and found that FGFR2 activation itself was not significantly affected by any of these mutations, indicating that the introduced mutations do not interfere with FGF7–FGFR2 interaction and activation. In summary, our data suggest that the changes in MAPK phosphorylation are most likely downstream consequences of the phosphorylated FGFR2 and independent of its phosphorylation status and suggest that FGF7/FGFR2-induced MAPK activation requires ADAM17-mediated release of the EGFR ligand HB-EGF.

### 3.5. Transformed Colony Formation in FGFR2-Expressing NIH 3T3 Cell Lines Depends on ADAM17 Activation

**Figure 6:**
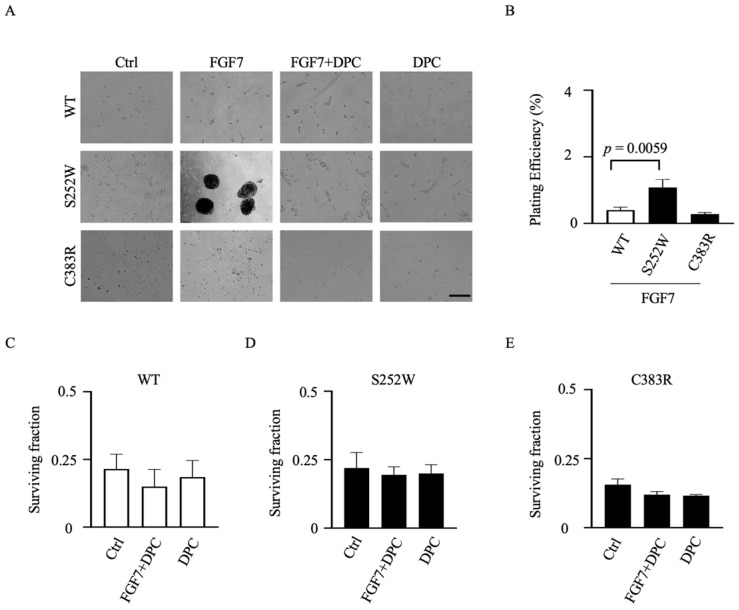
Analysis of FGF7/FGFR2-induced oncogenic growth of NIH 3T3 cells overexpressing mutant forms of FGFR2 in vitro. (**A**) Anchorage-independent growth was assessed by colony formation in soft agar. NIH 3T3 cells overexpressing the S252W mutant, along with wild type (WT) FGFR2- and FGFR2 (C383R)-overexpressing NIH 3T3 cells, were subjected to treatment with or without FGF7 (50 ng/mL) in the presence or absence of DPC333 (DPC; 2.5 μM), as indicated. Images were taken after two weeks (scale bar: 500 μm). (**B**–**E**) Plating efficiency (PE; number of colonies formed/number of cells seeded * 100%) (**B**) and surviving fraction (SF; number of colonies formed after treatment/number of cells seeded * PE * 100%) (**C**–**E**) of cells used in (**A**). Three independent experiments performed in duplicate. Colony counts from 10 random microscope fields in each replicate were analyzed for % plating efficiency (**B**) and survival fraction (**C**–**E**). Data are expressed as mean ± SEM; one-way ANOVA with Dunnett’s test. The *p* value indicates significantly increased plating efficiency of FGF7-treated NIH 3T3 cells overexpressing the S252W mutant cells compared with wild type (WT) FGFR2-overexpressing NIH 3T3 cells.

To gain further insights into the impact of gain- and loss-of-function mutant FGFR2 on oncogenic cell growth, we conducted clonogenic assays to assess the potential for anchorage-independent growth. Specifically, we compared the loss-of-function mutant FGFR2 (C383R) with the gain-of-function mutant FGFR2 (S252W) in NIH 3T3 cells, which have clonogenic potential unlike COS7 cells. Concordant with a key role for FGF7-driven oncogenicity, untreated FGFR2-mutant-expressing NIH 3T3 cells formed only a few colonies, but treatment with FGF7 led to a significant increase in colony-forming activity within two weeks (Figure 6A). Conversely, even in the presence of FGF7, NIH 3T3 cells expressing FGFR2-WT did not exhibit colony formation. Since ADAM17 is the subsequent step following FGFR2 activation in migration and proliferation of EC cells [25], targeting ADAM17 function in FGFR2-mutant cells may have a beneficial effect in tumor suppression. To examine the therapeutic potential, we assessed colony formation in the presence of the metalloprotease inhibitor DPC333 (Figure 6A, a quantification of the results of three separate experiments is shown in Figure 6B–E). We found that colony formation of *FGFR2*-mutant cells was sensitive to DPC333 treatment, indicating that blockade of ADAM17 activity significantly inhibited anchorage-independent growth in *FGFR2*-mutant cells.

## 4. Discussion

*FGFR2* gene mutations, which can be found in up to 25% of ECs [25] and are present in different regions of the FGFR2 protein [6], have previously been shown to drive cellular proliferation, survival, and other oncogenic processes [9]. As such, FGFR2 is an attractive therapeutic target [7,8,9,10,11]. However, the clinical responses to FGFR inhibitors are variable [14], underscoring the importance of elucidating whether all FGFR2 alterations are oncogenic and suitable for effective treatment with FGFR2 inhibitors. In this study, we have exploited an HB-EGF shedding assay as a screening method to investigate the functional consequences of *FGFR2* mutations in EC. This assay represents a significant advancement in personalized treatment approaches for cancers with *FGFR2* mutations, as the existing characterization methods have been limited by their lack of specificity and high-throughput capabilities [36]. The utilization of the HB-EGF shedding assay provides a robust and efficient alternative to conventional low-throughput methods, such as immunoprecipitation and phospho-specific antibody arrays, enabling the accurate evaluation of FGFR2 stimulation through assessing the activation of the metalloprotease ADAM17. Through the utilization of the HB-EGF shedding assay, we found that none of the tested mutations, which are commonly found in ECs, affected the constitutive activity of ADAM17. This suggests that the oncogenic effects of mutant FGFR2, particularly in relation to ADAM17 activation, are reliant on stimulation by the FGF7 agonist.

Upon stimulation with FGF7, we identified five *FGFR2* mutations (D101Y, S252W, I548V, N550K, and K660E), including three previously uncharacterized mutations, that exhibited a coupling with ADAM17, resulting in increased ADAM17-dependent AP-HB-EGF release. Additionally, we observed that K310R and C383R *FGFR2* mutations lacked the ability to activate ADAM17. Importantly, the absence of activation in C383R FGFR2-expressing cells was associated with a decreased capacity for anchorage-dependent growth in vitro. These findings underscore the utility of the HB-EGF shedding assay as a powerful tool for comprehensive characterization of *FGFR2* mutations. Importantly, our results suggest that *FGFR2* mutations can lead to both loss-of-function and gain-of-function outcomes, specifically in the context of ADAM17-dependent MAPK activation.

Notably, we observed that mutations in the juxtamembrane and transmembrane domains of FGFR2 were associated with a reduction or loss of ADAM17 and MAPK activation, while a mutation in the extracellular domain significantly increased the sensitivity of FGF7 ligand-induced ADAM17-mediated shedding of HB-EGF compared to cells expressing the WT FGFR2. Although the mechanism by which loss-of-function mutations fail to exert downstream activity is still unclear, it is noteworthy that the transmembrane domain of ADAM17 is required for its activation by various physiological stimuli [31]. Moreover, the juxtamembrane and transmembrane domains of ADAM17 and the seven-membrane-spanning inactive rhomboid protein 2 (iRhom2) have been implicated in the regulation of ADAM17 function [37]. iRhom2, also known as Rhbdf2, plays a crucial role in regulating the activity and substrate specificity of ADAM17. As a member of the rhomboid family of proteins, iRhom2 has emerged as a key player in the proteolytic processing of various transmembrane proteins [38]. Its function involves the formation of heterotrimeric complexes with ADAM17 and its substrates in the endoplasmic reticulum, facilitating their trafficking to the cell surface for subsequent proteolytic cleavage [39]. Exploring the potential functional interaction between the juxtamembrane and transmembrane domains of FGFR2, iRhom2, and ADAM17 could provide valuable insights into their activation mechanisms. Future studies focusing on this potential interaction hold great promise in unraveling the intricate regulatory mechanisms underlying ADAM17 activity and substrate specificity. This investigation has the potential to yield profound insights and may serve as a stepping stone for the development of innovative therapeutic approaches aimed at targeting FGFR2 signaling and addressing associated pathologies. Consequently, our results shed light on the posttranslational regulation of ADAM17’s proteolytic activity, emphasizing the significance of the juxtamembrane and transmembrane domains of FGFR2.

These findings also underscore the role of ADAM17 as a key integrator of the FGFR2 signaling pathway, which has been implicated in various cancer types [40,41,42,43]. This raises the possibility that several gain-of-function mutations in the *FGFR2* gene could contribute to tumor growth and the formation of metastasis in EC by regulating ADAM17-mediated MAPK signaling pathways.

Our investigation revealed that the tumor-driver potential of gain-of-function mutant FGFR2 depends on ADAM17-mediated MAPK activation, while the overexpression of loss-of-function *FGFR2* mutants showed only minimal tumorigenicity in the absence of other driver alterations in cell-based assays. These results support the concept that the oncogenic competence of mutant FGFR2 could rely on co-drivers such as ADAM17 and EGFR-mediated MAPK activation. Interestingly, previous studies have demonstrated that the oncogenic competence of full-length FGFR2 depends on co-drivers that potentially enhance canonical FGFR2 signaling [25,44]. In clinical trials, objective responses to FGFR inhibition were rarely observed in patients with FGFR2 amplification and fusion structural variants. However, patients with truncating FGFR2 variants exhibited favorable responses [44], while patients with other FGFR alterations showed less promising outcomes [45,46,47,48,49]. Hence, the efficacy of FGFR inhibitors may be influenced by the expression of specific *FGFR2* mutations compared to FGFR2 alterations that rely on oncogenic co-drivers.

## 5. Conclusions

In summary, our study provides significant insights into the functional distinctions among various *FGFR2* mutations in EC. These mutations have distinct effects on FGF7-induced release of HB-EGF and MAPK phosphorylation, highlighting the importance of specific genetic alterations in modulating downstream signaling pathways and their potential implications for the oncogenic activity of FGFR2 in the context of ADAM17 activation. These findings contribute to our understanding of the role of *FGFR2* mutations in EC and their suitability as targets for drug intervention. Moreover, our results suggest that the distribution of mutations within the *FGFR2* gene can influence their oncogenic effects, expanding our understanding of ADAM17 regulation and its interaction with FGFR2. Ultimately, the development of the HB-EGF shedding assay represents a significant advancement in our comprehension of FGFR2 signaling and its associated mutations. It provides a specific, high-throughput approach for studying FGFR2-related disorders, enabling more effective personalized treatment strategies for cancers harboring *FGFR2* mutations.

## Data Availability

Data are contained within the article.

## Abbreviations

ADAM17	A disintegrin and metalloprotease 17
AP	Alkaline phosphatase
EC	Endometrial cancer
EGFR	Epidermal growth factor receptor
FGF	Fibroblast growth factor
FGFR2	Fibroblast growth factor receptor 2
FRS2	Fibroblast growth factor receptor substrate 2
HB-EGF	Heparin-binding EGF-like growth factor
iRhom2	Inactive rhomboid protein 2
MAPK	Mitogen-activated protein kinase
